# The Full Active Principle in Drugs

**Published:** 1907-04-27

**Authors:** 


### Points in Treatment.

### THE FULL ACTIVE PRINCIPLE IN DRUGS.

### The Difficulty of being Certain that Drugs Prescribed or Used in Dispensing Contain the Full Amount of Active Principle.

We have already discussed the difficulty there is in knowing whether such drugs as digitalis, strophanthus, squill, and ergot contain the full amount of active principle; in the case of these drugs the trouble arises, not from adulteration, but from the fact that the active principles are not alkaloids but glucosides, are unsuited for chemical analysis, and can only be standardised by physiological experiment. There are large numbers of other drugs, however, which, for the sake of cheapness, are liable to adulteration. There is so great a tendency nowadays to buy things in the cheapest market that the quality of the drugs used in making up prescriptions runs a very great danger of deteriorating. The medical man has no time to test his drugs for himself; if he buys in the cheapest market, as most of us are naturally inclined to do, he lays himself open to be deceived in the quality of his drugs; and the curative effects of his treatment will be less good than those of his neighbour who takes more care to know that his drugs are pure.

It is well, therefore, to purchase medicines only from such firms as are known to take the greatest care in avoiding adulterated samples of the commercial drugs, even though the cost is necessarily a little more.

It is wonderful what trouble is taken by some firms in analysing the crude drugs. The amount of work entailed in determining specific gravities; saponification values; acidities; percentages of ash, resin, extractives; melting-points; solubilities in various strengths of alcohol; iodine absorptions; ester values; polarimetric angles of rotation; alkaloid percentages; and so on, is very great. It costs money, and the medical man cannot but be grateful to the chemists who do these things for him.

The following examples will suffice to show the importance of this. They are taken from the annual report of a large analytical laboratory.

*Sandal-wood* oil is an example of an expensive medicine which it well pays the producer to adulterate. It can be bought at all sorts of prices, but it becomes almost a certainty that the very cheap samples will fail to cure where dearer samples of known purity would greatly accelerate the recovery of the patient; for it is a medicine of undoubted efficacy when that which is given is really sandal-wood oil and not a fluid which merely bears that name. The report of the analyst says: "Considerable care is necessary before accepting parcels of this oil as genuine." Parry has shown that fractional distillates of the West Indian oil (from *Amyris balsamifera*) are used for sophistication, and altogether the "blending" of this product appears to have reached the stage of a science. This warning is the more necessary where the oil is sold in capsules, and hence less readily accessible for testing. A considerable number of the samples examined gave results varying within the following limits:—

| | | | |
|---|---|---|---|
| Specific gravity ... | 0.975 | to | 0.990 |
| Rotation ... ... | −15.75° | to | −19.5° |
| Santalol ... ... | 91.61 | to | 98.79 per cent. |

All soluble in 6 volumes of 70 per cent. alcohol.

As an example of the oils, necessarily styled "suspicious," is the following:—

| | |
|---|---|
| Specific gravity ... ... | 0.972 |
| Rotation ... ... ... | −16.10° |
| Santalol ... ... ... | 89.14 per cent. |

Not entirely soluble in 6 volumes of 70 per cent. alcohol.

*Copaiba*: "We have again to record our experience that a large proportion of the parcels of copaiba on the market give reactions more or less distinct when the colour tests are applied. Of the eleven samples submitted to examination, the results of which are recorded below, but one could be described as giving no reaction with these tests, while in no case could gurjun balsam be detected according to the indications of the resin acid factor."

| | | | |
|---|---|---|---|
| Specific gravity ... | 0.9679 | to | 1.0108 |
| Resin ... ... ... | 36.4 | to | 64.9 per cent. |
| Acid figure ... ... | 47.44 | to | 91.81 |
| Ester figure ... ... | 3.0 | to | 8.7 |
| Resin acid factor ... | 0.61 | to | 0.79 |

Another sample of balsam was expressed from capsules offered at an exceptionally low price, and gave results which would appear to indicate gross adulteration. They are as follows:—

| | |
|---|---|
| Specific gravity ... ... ... | 0.9507 |
| Resin (soft in character) ... | 42.22 per cent. |
| Acid figure ... ... ... | 35.8 |
| Ester figure ... ... ... | 17.0 |
| Resin acid factor ... ... | 1.185 |

Official colour tests, deep purple.

*Jalap*: "Seventeen samples of jalap have been assayed for resin, no one of which satisfied the official standard, the average figure obtained being the worst we have yet met with. Total resin 3.8 to 8.4, averaging 6.3, per cent."

These three examples have been picked out because they concern common drugs which are of known efficacy when they are pure. It must not be supposed that there is an all-round adulteration of every drug upon the market; the report of the same analytical laboratory contains scores of analyses of drugs which have proved to be quite pure. The point is that, without the check of repeated analysis of samples, an article may be called by such-and-such a name, and sold accordingly, when its quality, and consequently its curative action, is very much below par. However much one may be tempted to buy the cheapest samples possible, one should refrain from doing so unless, in addition to being cheap, the medicine has some guarantor that it is pure.

